# Cyclophilin Inhibitor NV556 Reduces Fibrosis and Hepatocellular Carcinoma Development in Mice With Non-Alcoholic Steatohepatitis

**DOI:** 10.3389/fphar.2019.01129

**Published:** 2019-09-26

**Authors:** Joseph Kuo, Sonia Simón Serrano, Alvar Grönberg, Ramin Massoumi, Magnus Joakim Hansson, Philippe Gallay

**Affiliations:** ^1^Department of Immunology & Microbiology, The Scripps Research Institute, La Jolla, CA, United States; ^2^NeuroVive Pharmaceutical AB, Lund, Sweden; ^3^Department of Laboratory Medicine, Translational Cancer Research, Lund University, Lund, Sweden

**Keywords:** histology, inflammation, cytokines, steatosis, tumor burden

## Abstract

Hepatocellular carcinoma (HCC), the third major cause of cancer mortality, can result from non-alcoholic steatohepatitis (NASH). Due to limited efficacy of drugs approved for HCC and no drug available yet for NASH, identification of new effective treatments is crucial. Here, we investigated whether NV556, a cyclophilin inhibitor derived from sanglifehrins, would decrease the development of NASH and HCC in a preclinical mouse model. For our experiment, male mice were administered streptozotocin to disrupt pancreatic cells and nourished with high-fat diet since 3 weeks of age. Afterward, NV556 or vehicle was orally administered daily for 6 weeks before the 14-week-old time point for the development of NASH, or 10 weeks before the 30-week-old time point for the establishment of HCC. Body weight, blood glucose level, and liver weight were recorded. Moreover, for NASH, livers were histologically examined for inflammation and steatosis. Collagen was measured by Sirius Red staining of hepatic tissues. Systemic cytokine levels in serum were detected by multiplex assays. For HCC, nodules of livers were measured and scored according to a developed system with number and size of nodules as criteria. NV556 significantly decreased collagen deposition (*p* = 0.0281), but did not alter inflammation, steatosis, body and liver weight, and systemic cytokine production compared to control mice with NASH symptoms. For HCC, NV556 statistically reduced the number (*p* = 0.0091) and diameter of tumorous nodules (*p* = 0.0264), along with liver weight (*p* = 0.0026) of mice.Our study suggests NV556 as a promising candidate for treatment of NASH-derived fibrosis and HCC.

## Introduction

Hepatocellular carcinoma (HCC) is the third major cause of cancer mortality, globally accounting for 800,000 deaths per year (Flores and Marrero, 2014). Moreover, HCC is one of the fastest-growing causes of cancer mortality in the United States (Flores and Marrero, 2014). Current treatment for HCC includes liver transplantation, segmentectomy, chemotherapy, and systemic drug therapy. However, tumor recurrence may occur after transplantation and segmentectomy, and HCC is minimally responsive to chemotherapy. Moreover, chemotherapy presents numerous toxic side effects (Llovet and Bruix, 2008; Bruix and Sherman, 2011). Importantly, unexpectedly high rates of HCC recurrence occur after hepatic resection (Pravisani et al., 2017) and chemotherapy (Conti et al., 2016; Reig et al., 2016). Therefore, the identification of new effective treatments for HCC is of a crucial research interest.

HCC is not only caused by alcoholic cirrhosis and viral hepatitis, but also caused by non-alcoholic fatty liver disease (NAFLD) (White et al., 2012; Flores and Marrero, 2014). NAFLD, the hepatic manifestation of metabolic syndrome, is now the most common liver disorder in the Western world due to the rise in obesity and diabetes (Bellentani, 2017). NAFLD is a complex disease, beginning with benign, asymptomatic accumulation of fat droplets in hepatocytes to the more aggressive, inflammatory stage of non-alcoholic steatohepatitis (NASH). Patients with NASH are at increased risk to progress to HCC (White et al., 2012). Moreover, NASH, with increasing prevalence, is projected as the leading cause of liver transplantation in the United States and developing countries within the next decade (Charlton et al., 2011; Agopian et al., 2012). Importantly, no evidence-based drug therapy has been approved to treat NASH, classifying NASH as a medical condition with high, unmet therapeutic necessity.

What could drive the progression from NAFLD to NASH and further to HCC? Cyclophilins (Cyps), a family of ubiquitous cellular proteins that includes CypA and CypB, may contribute to disease progression of viral hepatitis infections, cancer, and other inflammatory diseases (Yang et al., 2008; Satoh et al., 2009; Nigro et al., 2013). Importantly, HCC tissues from human patients have been shown to overexpress CypA, which promotes growth of tumorous cells and is associated with HCC progression and metastasis (Zhang et al., 2011; Gong et al., 2017). Overexpression of CypB was also reported in human HCC tissues compared to normal liver tissues and protects hepatoma cells from oxidative stress (Kim et al., 2011; Kim et al., 2012). Hence, inhibition of Cyps may decrease the progression from NAFLD to NASH and HCC. To date, no published study on inhibition of Cyps has been completed in NASH and HCC.

NV556 is a non-immunosuppressive cyclophilin inhibitor (CypI) formerly known as NVP018 and BC556 (Hansson et al., 2015) and is a derivate of sanglifehrin (Sf). Sf, produced by soil *Streptomyces* bacteria, is a compound that binds to Cyps and inhibits cyclophilin activities (Zydowsky et al., 1992; Zenke et al., 2001; Sedrani et al., 2003). However, Sf has some immunosuppressive activity and poor drug properties, which is not ideal for targeting chronic diseases. Therefore, non-immunosuppressive CypI of Sf analog has been created and studied (Gregory et al., 2011; Hansson et al., 2015). Moreover, similar to other CypIs, NV556 does not affect the expression of cyclophilins but bind to hydrophobic pockets of cyclophilins. Therefore, the enzymatic activities of cyclophilins would be neutralized, creating an inhibitory effect (Gregory et al., 2011; Hansson et al., 2015).

In this study, we aimed to examine whether administration of CypI NV556 would decrease the development of NASH and HCC in a preclinical murine model, with symptoms established in months compared to years and decades in human patients. Initially, 2-day-old male C57BL/6J mice were intraperitoneally injected with a single dose of streptozotocin (STZ), an antibiotic that is toxic to pancreatic insulin-producing β cells and nourished with high-fat diet (HFD) to induce steatosis (Fujii et al., 2013). We hypothesized that experimental mice receiving NV556 have decreased NASH and HCC progressions compared to vehicle control. Our results showed that blockade of Cyps with NV556 reduced the development of NASH-related fibrosis and tumorous nodules in mice.

## Materials and Methods

**Mice.** Experimental protocols were approved by the Institutional Animal Care and Use Committee of The Scripps Research Institute (La Jolla, CA) and adhered to guidelines from the National Institutes of Health (Bethesda, MD). Female pregnant E14 C57BL/6J mice were purchased from the Department of Animal Resource’s Rodent Breeding Colony (The Scripps Research Institute; La Jolla, CA), with pups used for experimentation. All mice were kept at the vivarium in The Scripps Research Institute’s Department of Immunology & Microbiology (La Jolla, CA). Mice were maintained under a pathogen-free condition at 21°C with food and water provided *ad libitum* during daily cycles of 12 h of light and darkness.

**Induction of NASH and HCC**. After birth, 2-day-old C57BL/6J male pups were selectively injected intraperitoneally with 200 μg of STZ (Sigma-Aldrich; St. Louis, MO) to disrupt pancreatic β cells, induce diabetes, and promote steatosis in the liver. Females were excluded since male C57BL/6J mice were reported with more susceptibility to STZ-induced diabetes (Nakamura et al., 1984), HFD-mediated hyperglycemia (Asha et al., 2016), and chronic inflammation (Morselli et al., 2014). Moreover, C57BL/6J murine strain was used since other strains are shown with more resistance to STZ-induced diabetes, adiposity, liver inflammation, and fibrosis from HFD compared to C57BL/6J mice (Gurley et al., 2006; Jovicic et al., 2015). Sex of mice was confirmed at 1 week old for experimentation. At 3 weeks old, mice were weaned and received HFD with 60%kcal fat (Product D12492N from Research Diets Inc., New Brunswick, NJ). In this model, NASH and HCC are sequelae of hyperglycemia from diabetes and obesity due to hepatocellular stress, inflammation, degeneration of hepatocytes, apoptosis, and fibrosis (Fujii et al., 2013). Mice and diet were maintained until the detection of NASH at 14 weeks old and HCC at 30 weeks old. Additionally, mice under negative control diet (Product D12450KN from Research Diets Inc., New Brunswick, NJ) and without STZ exposure were included.

**Figure 1:**
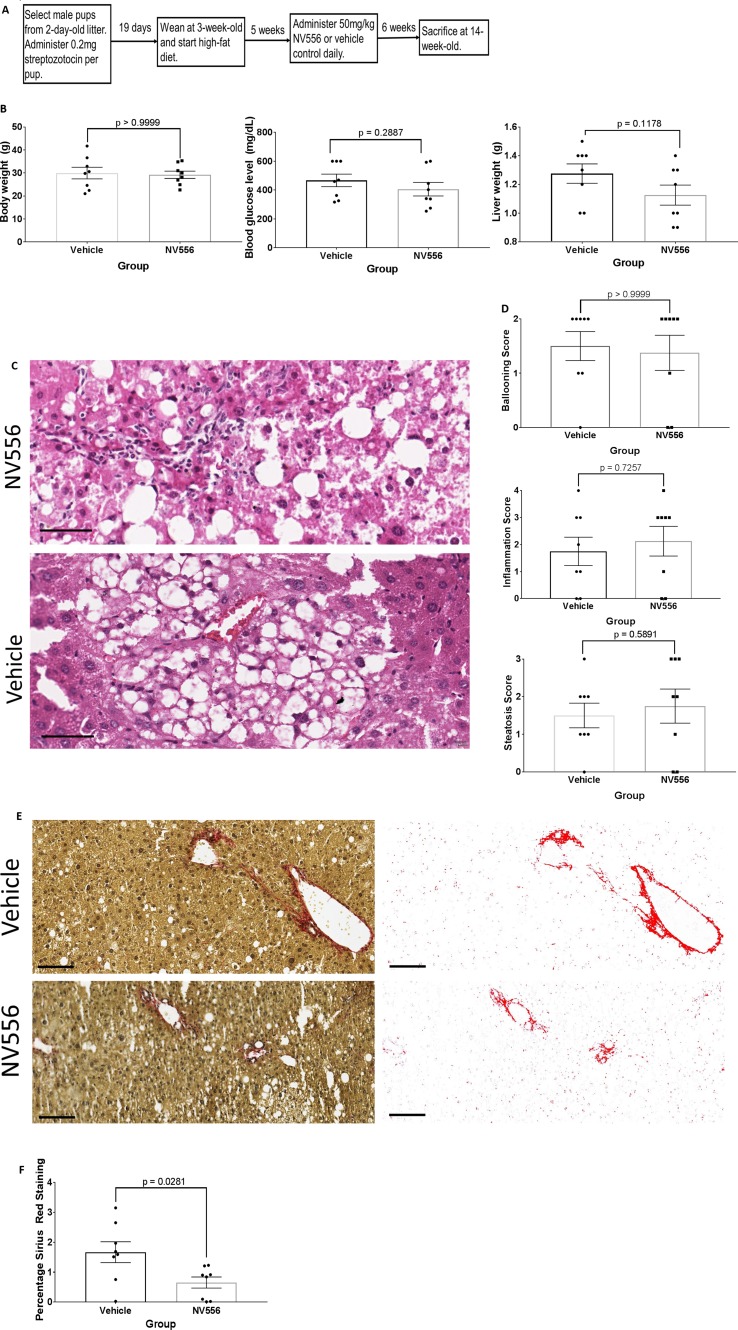
NV556 reduced liver fibrosis at NASH timepoint. **(A)** Flowchart for studies on NASH and fibrosis. Fibrosis was induced in male C57BL/6J mice by intraperitoneal injection of 200 µg of STZ 2 days after birth followed by a free-feeding high-fat diet (60% kcal fat) after 3 weeks of age for 11 weeks. 50mg/kg NV556 or vehicle control was administered via oral gavage daily to mice for 42 days before sacrifice at 14 weeks of age. **(B)** Body weight (left), blood glucose level (middle), and liver weight (right) of mice. **(C)** Hematoxylin and eosin (H&E) staining on paraffin-embedded liver sections. Bar indicates 50μm. **(D)** Ballooning, inflammation, and steatosis scoring on H&E stained tissues. **(E)** Liver fibrosis identified by Sirius red staining (left) and quantified by ImageJ (right). Bar indicates 100μm. **(F)** Percentage of Sirius Red staining. All error bars indicate ± standard error from the mean, with p-values from two-tailed Mann-Whitney test.

**Administration of NV556.** NV556 was kindly provided by Neurovive Pharmaceutical AB (Lund, Sweden). Vehicle contained 5% absolute ethanol, 5% Cremophor EL (Sigma-Aldrich; St. Louis, MO), and 90% sterile saline solution (Teknova; Hollister, CA). STZ-injected and HFD-nourished C57BL/6J male mice were randomly assigned to receive NV556 or vehicle control for NASH (*n* = 8 per treatment) or HCC (*n* = 10 per treatment) study (**Figures 1A** and **3A**). More specifically, to examine the effect of NV556 on NASH, 8-week-old mice were treated with vehicle or 50 mg/kg NV556 in a 0.1-ml volume via oral gavage daily for 42 days until 14 weeks old. For HCC, vehicle or 50 mg/kg NV556 in 0.1-ml volume was administered to 20-week-old mice via oral gavage for 10 weeks until 30 weeks old. Mice were anesthetized with 1% isoflurane before oral gavage to decrease pain and discomfort.

**Measurement of body and liver weights and detection of blood glucose level.** For both NASH and HCC studies, mice were fasted for 6 h before experimental end points. Then, mice were placed in an anesthesia induction chamber with 2% isoflurane until unconscious, followed by body weight measurement with a digital balance scale (Ohaus Corporation; Pine Brook, NJ) with precision of 0.1g. Afterward, mice were anesthetized with 2% isoflurane contained in a nose-and-mouth cup, with whole blood from each mouse drawn via cardiac puncture with a 27-gauge needle and syringe coated with heparin. Fifty microliters of blood per mouse was then detected for blood glucose level with OneTouch Ultra2 Meter (LifeScan, Inc., Milpitas, CA) and suited test strips (PharmaTech Solutions, Inc., Westlake Village, CA). Lastly, mice were euthanized with isoflurane, and entire livers were harvested and weighed individually.

**Figure 2:**
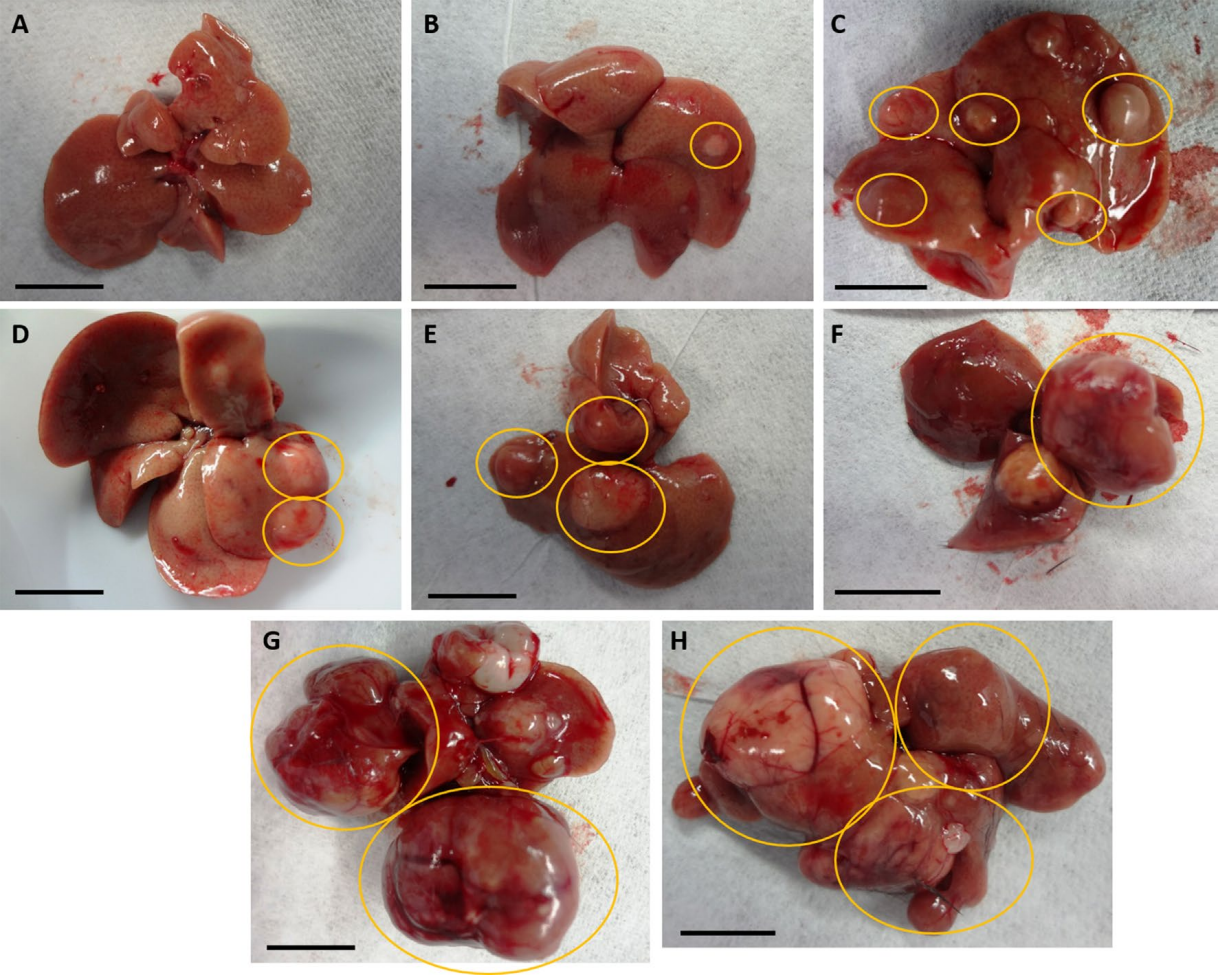
Representations of nodular livers with tumor burden scoring. Diameter and numbers of detectable nodules in entire livers of mice were considered for scoring from 0 to 7, as seen in Table 1. **(A)** No detectable nodules; score of 0. **(B)** Only and no more than 4 small nodules; score of 1. **(C)** More than 4 small detectable nodules, with no larger nodules; score of 2. **(D)** Less than 3 medium-sized nodules and no larger nodules; score of 3. **(E)** 3 or more medium-sized nodules with no larger nodules; score of 4. **(F)** No more than 1 large nodule; score of 5. **(G)** No more than 2 large nodules; score of 6. **(H)** 3 or more large nodules; score of 7. Nodules in yellow circles indicate those fitting each scoring criterium.

**Determination of tumor burden.** Recent murine studies on HCC showed number of tumors separately from maximal tumor size or tumor area (Nakagawa et al., 2014; Casagrande et al., 2017; Van der Windt et al., 2018). Here, we developed a tumor burden scoring system that includes both number and diameter of visible nodules at the outer surface of livers (**Table 1** and **Figure 2**). Hepatic lobes were examined for tumorous nodules, especially for the HCC study, with number and diameter to the nearest millimeter of nodules in the entire liver recorded. In our established scoring system for tumor burden, nodules were considered detectable with a diameter of >0.1 cm. Nodules with a diameter not exceeding 0.5 cm were categorized as small, whereas that of medium-sized nodules was less than 1.0 cm. Nodules with a diameter ≥1.0 cm were considered large. Ratings included the following: 0 (no detectable nodules), 1 (no more than four small nodules with no larger nodules), 2 (more than four small nodules with no larger nodules), 3 (no more than two medium-sized nodules with no larger nodules), 4 (three or more medium-sized nodules with no larger nodules), 5 (no more than one large nodule), 6 (no more than two large nodules), and 7 (three or more large nodules). This system with scores from 0 to 7 was established to be more comprehensive than previous studies (Nakagawa et al., 2014; Casagrande et al., 2017; Van der Windt et al., 2018) since tumors of different sizes may be detected in entire livers.

**Table 1:** Tumor burden scoring system of nodules in livers.

Numerical Score	Description
0	No detectable nodules
1	Only and no more than 4 small (diameter ≤ 0.5 cm) nodules
2	More than 4 small detectable nodules, with no larger nodules
3	Less than 3 medium-sized (diameter > 0.5 and <1.0 cm) nodules and no larger nodules
4	3 or more medium-sized nodules with no larger nodules
5	No more than 1 large (diameter ≥ 1.0 cm) nodule
6	No more than 2 large nodules
7	3 or more large nodules

Entire livers of mice were examined for diameter and number of nodules as signs of HCC. Nodules were considered detectable with a diameter of >0.1 cm. Nodules with diameter not exceeding 0.5 cm were categorized as small, whereas that of medium-sized nodules was less than 1.0 cm. Nodules with diameter ≥ 1.0 cm were considered large. The score of 0 to 7 depends on the number of nodules from each category.

**Histological examination.** After harvest, livers were fixed in buffered zinc formalin solution (StatLab Medical Products, McKinney, TX) for at least 72 h. Livers were then processed, embedded in paraffin, and sectioned into five to ten 6-μm-thick sections. Each section of the livers was placed onto a glass slide. One set of slides was stained with hematoxylin and eosin, while another was stained with Sirius Red (Puchtler et al., 1973; Junqueira et al., 1979). Slides were scanned with Aperio AT2 Digital Pathology Slide Scanner (Leica Biosystems Inc., Buffalo Grove, IL). Histopathological examinations of the tissues were conducted with guidance from a board-certified pathologist for ballooning, inflammation (including lobular and portal), and steatosis. The following ratings for NAFLD activity (Kleiner et al., 2005) were used for ballooning: 0 (no histopathological changes), 1 (few balloon cells), and 2 (many prominent balloon cells); for inflammation: 0 (no histopathological changes), 1 (minimal infiltration of leukocytes with no major inflammatory foci in the liver), 2 (mild with <2 inflammatory clusters), 3 (moderate with 2–4 foci of leukocytes), and 4 (severe infiltration of leukocytes with >4 inflammatory clusters); for steatosis: 0 (<5% steatosis), 1 (5–33%), 2 (33–66%), and 3 (>66% steatosis). Percentage of Sirius Red staining was quantified by ImageJ software (National Institutes of Health, Bethesda, MD).

**Quantification of systemic cytokine levels.** Plasma was isolated from whole blood of mice at time of sacrifice. Twenty-five microliters of plasma per mouse was used for a customized multiplex (U-PLEX) assay kit (Meso Scale Diagnostics, Rockville, MD) to detect levels of murine interferon gamma (IFNγ), interleukin (IL)-1 beta (IL-1β), IL-4, IL-6, IL-10, IL-15, IL-17A, IL-27, IL-33, tumor necrosis factor alpha (TNF-α), IFNγ-induced protein (IP)-10, macrophage inflammatory protein (MIP)-1 alpha (MIP-1α), and MIP-2. Cytokine levels were detected by MESO QuickPlex SQ 120 imager (Meso Scale Diagnostics, Rockville, MD) and read with the Discovery Workbench software (Meso Scale Diagnostics, Rockville, MD).

**Statistics**. Body weight, liver weight, blood glucose level, histological scores, percentage of Sirius Red staining, systemic cytokine levels, number of tumorous nodules, and nodular scoring among different groups of mice were evaluated by two-tailed Mann–Whitney nonparametric test through GraphPad Prism (GraphPad Software; San Diego, CA). Alpha level was set at 0.05. Data are presented as the average with standard error of the mean. Average IC_50_ from cell growth inhibition was obtained via transformation and non-linear regression, with *p* values and 95% confidence intervals from Kruskal–Wallis test through GraphPad Prism.

## Results

**NV556 reduces NASH-related liver fibrosis but not local inflammation.** The objective is to determine if NV556 would affect the development of NASH and fibrosis. Specifically, STZ-injected and HFD-nourished mice were administered vehicle (*n* = 8) or 50 mg/kg NV556 (*n* = 8) daily via oral gavage for 42 days starting at 8 weeks of age until 14 weeks old (**Figure 1A**); time point was selected due to the occurrence of NASH and fibrosis from our preliminary studies (data not shown). NV556 did not significantly alter body weight, blood glucose level, and liver weight of 14-week-old mice (**Figure 1B**). Moreover, histological scores of inflammation, steatosis, and ballooning were comparable between mice administered NV556 or vehicle control (**Figures 1C, D**). Conversely, the percentage of Sirius Red staining, indicative of collagen and fibrosis, was significantly reduced from 1.66 ± 0.34 in vehicle control mice to 0.65 ± 0.18 in NV556-treated experimental mice (**Figures 1E, F**). Naïve negative control mice administered vehicle control or NV556 had comparable liver weight, lower body weight (15–25 g), and no histological changes (data not shown). Moreover, no side effects were observed.

**NV556 does not affect systemic cytokine levels.** With decreased Sirius Red staining and comparable scoring of localized hepatic inflammation, we questioned if NV556 would alter systemic inflammation. Studies have associated the induction of hepatitis with innate immunity and acquired type 1 pro-inflammatory response including TNF-α, IL-1β, IL-6, and IL-12 (Zydowsky et al., 1992; Zenke et al., 2001; Sedrani et al., 2003). Recently, Hart et al. (Hart et al., 2017) showed that type 2 immunity may contribute to the exacerbation of NAFLD with transforming growth factor beta (TGFβ). Other studies also showed the potential role of pro-inflammatory IL-17-producing T helper 17 cells in NASH development (Giles et al., 2015; Rau et al., 2016). Therefore, we measured levels of an array of cytokines in plasma of experimental mice with or without NV556. Surprisingly, levels of all 13 examined cytokines from plasma of NV556-treated experimental mice were insignificantly (*p* > 0.05) different from vehicle control (**Supplementary Figure 1**).

**NV556 decreased body weight, liver weight, tumor burden, hepatic inflammation, and fibrosis in mice with NASH-induced HCC.** The objective is to determine if NV556 would affect the development of NASH-induced HCC. We developed a scoring system where both number and diameter of tumor nodules are considered (**Table 1** and **Figure 2** showing representative livers from the study). This system is more reflective of tumor burden instead of reporting either the number or size of tumors from previous studies (Nakagawa et al., 2014; Casagrande et al., 2017; Van der Windt et al., 2018).

**Figure 3:**
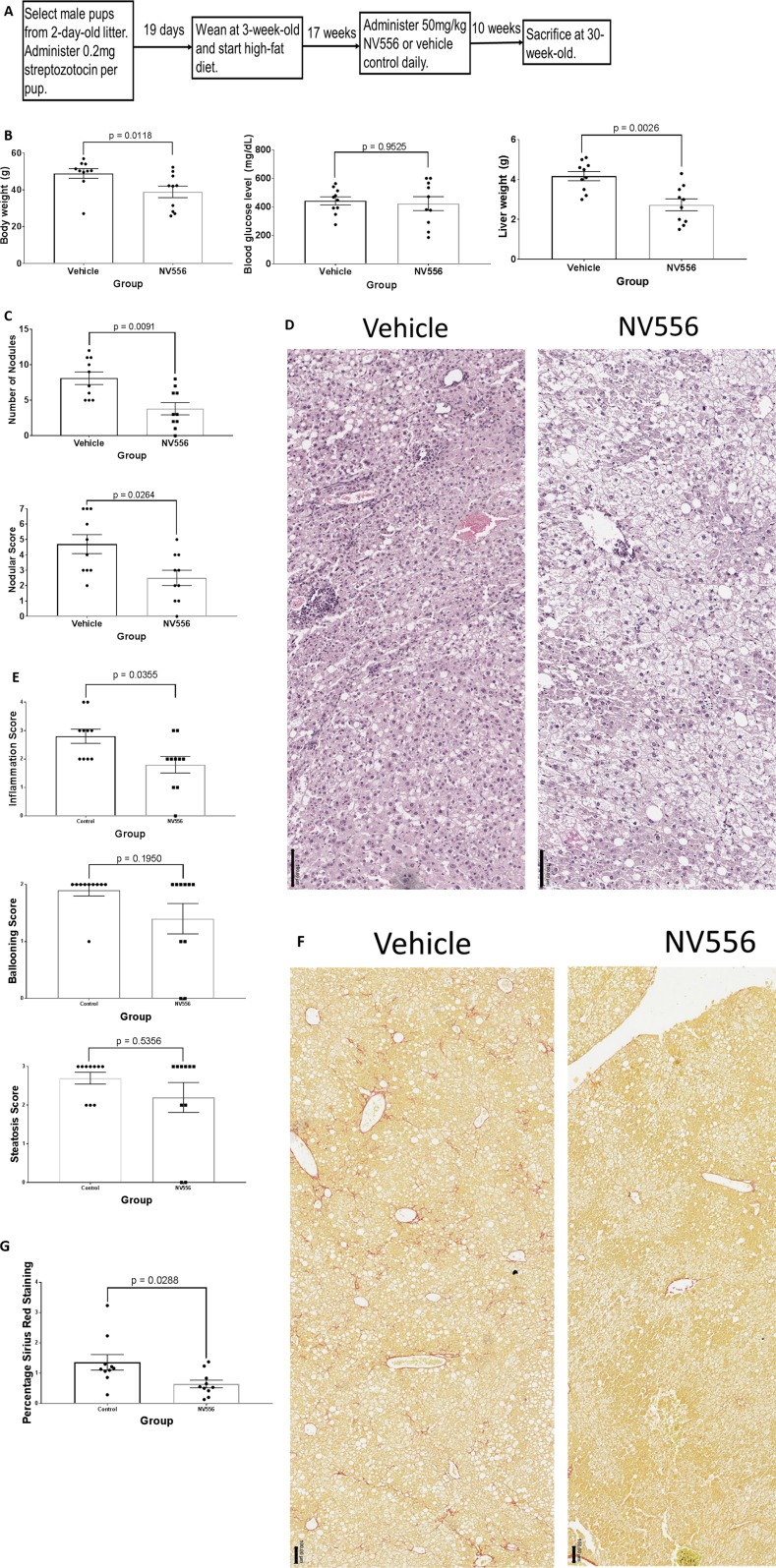
NV556 decreased body weight, liver weight, tumor burden, inflammation, and fibrosis in mice with NASH-induced HCC. **(A)** Flowchart for studies on HCC. HCC was induced in male C57BL/6J mice by intraperitoneal injection of 200 µg of STZ 2 days after birth followed by a free-feeding high-fat diet (60% kcal fat) after 3 weeks of age for 27 weeks. Fifty mg/kg NV556 or vehicle control was administered via oral gavage daily to mice for 70 days before sacrifice at 30 weeks of age. **(B)** Body weight (left), blood glucose level (middle), and liver weight (right) of mice. **(C)** Number of detectable nodules (left) and nodular scoring for tumor burden (right) in livers of mice. **(D)** H&E staining on paraffin-embedded liver sections. Bar indicates 50μm. **(E)** Inflammation, steatosis, and ballooning scoring on H&E stained tissues. **(F)** Liver fibrosis identified by Sirius red staining (left) and quantified by ImageJ (right). Bar indicates 100μm. **(G)** Percentage of Sirius Red staining. All error bars indicate ± standard error from the mean, with p-values from two-tailed Mann-Whitney test.

STZ-injected and HFD-nourished 20-week-old mice were administered vehicle (*n* = 10) or 50 mg/kg NV556 (*n* = 10) daily for 70 days until sacrifice at 30 weeks of age (**Figure 3A**); no tumorous nodule was observed in experimental and control mice at 20 weeks of age, and the 30-week-old time point was selected due to evident hepatomegaly and tumors from preliminary studies (data not shown). NV556 significantly decreased body and liver weight of STZ-injected, HFD-nourished mice with NASH-driven HCC (**Figure 3B**). Blood glucose level was not affected (**Figure 3B**). The number of nodules also significantly decreased from 8.10 ± 0.88 in vehicle control mice to 3.80 ± 0.86 in NV556-treated mice (**Figure 3C**). Nodular scoring, which includes diameter of all nodules into consideration, was also significantly lowered from 4.70 ± 0.61 in vehicle control mice to 2.50 ± 0.50 in NV556-treated mice (**Figure 3C**). Naïve negative control mice administered vehicle control or NV556 had liver weight in the range of 1.0–1.6 g, body weight between 20 and 40 g, no histological changes, and no tumorous nodules (data not shown). No side effects were observed.

Though liver weight decreased in NV556-treated mice, histological scores of steatosis and ballooning were comparable to vehicle control mice (**Figures 3D, E**) when examining stained hepatic regions with tumors, if detected. In contrast, inflammation score was significantly (*p* = 0.0355) reduced in NV556-treated mice. The percentage of collagen fibers stained with Sirius Red also significantly (*p* = 0.0288) decreased with NV556 treatment (**Figures 3F, G**).

## Discussion

Cyps, a family of ubiquitous cellular proteins, have been shown to activate T lymphocytes, stimulate pro-inflammatory cytokine signaling, and recruit leukocytes in viral infections, cancer, and other human diseases (Nigro et al., 2013; Dawar et al., 2017). Moreover, Cyps were reported to inhibit CD4^+^ T helper 2 cell differentiation, therefore promoting T helper 1 cell proliferation (Colgan et al., 2004). Regarding viral hepatitis, we previously showed that various CypI, such as non-immunosuppressive cyclosporine analogs, the other structurally distant class of CypI, block hepatitis B and C viral infections and activities (Baugh and Gallay, 2012; Chatterji et al., 2014; Gallay et al., 2015; Philips et al., 2015; Gallay et al., 2019). The Sf analog NV556 also inhibits HBV and HCV infections (Gregory et al., 2011; Hansson et al., 2015).

Regarding tumor development, Volker *et al.* (Volker et al., 2018) showed that Cyps promote mammary tumorigenesis in mice. In a non-small cell lung cancer murine model, overexpression of Cyps elevated tumor metastasis rate compared to those with downregulated Cyps (Guo et al., 2018). Regarding NAFLD, levels of CypA, a member of the cyclophilin family were elevated in serum of human patients, especially in those with high glucose levels (Mutlu et al., 2017). Overexpressed CypA levels were also observed in human HCC tissues, with CypA promoting cell cycle from G1 to S phase (Yang et al., 2008). Moreover, elevated CypB level was also detected in human HCC tissues compared to normal liver tissues (Kim et al., 2011; Kim et al., 2012).

Similarly, our results in this study supported the pro-tumorigenic role of Cyps, where inhibition of Cyps with NV556 decreased tumor burden in mice with NASH-driven HCC. Although steatosis was still present in NV556-treated mice at the HCC time point, reduction of body and liver weight in these mice could be attributed to the decrease in tumorous nodules and tumor burden. Interestingly, our data suggested an anti-fibrotic effect of NV556 in the hepatic cellular matrix, as shown in both NASH and HCC time points. Since fibrosis is associated with inflammation, we expected decreased inflammation with NV556 treatment. However, our data show that NV556 did not affect the inflammation score at the NASH time point, arguing against an anti-inflammatory mechanism behind the anti-fibrotic effect. Since steatosis was still evident after NV556 treatment, persisting lipotoxicity due to adipose deposit from HFD intake may continually induce ballooning and proinflammatory signals from other signaling molecules despite inhibition of Cyps. At the HCC time point, inflammation scoring of vehicle control mice was higher compared to the NASH time point. Without any increase in fibrosis, inflammation could be attributed to tumor growth. Since NV556 inhibited tumor growth, HCC-dependent inflammation would therefore be reduced. Further studies to determine mechanisms of action with biomarkers, gene expressions, and liver enzyme levels are underway.

Our murine model with STZ and HFD administration is also one of the many NASH-driven animal models for clinical trials and has its own caveats compared to human patients. First, female mice in our model have been reported to not exhibit NASH symptoms, perhaps due to estrogen levels (Nakamura et al., 1984; Morselli et al., 2014; Asha et al., 2016). However, female human NASH and NASH-driven HCC patients exist (Yang et al., 2014). Second, the range of body weight was larger at the earlier NASH time point, mostly due to HFD provided ad libitum with different HFD intake among mice and was not as narrow as previously reported (Fujii et al., 2013). However, mice at the HCC time point were overweight and obese with narrower range of body weight due accumulation of adipose deposit in visceral masses. In contrast, not all human patients are overweight or obese (Margariti et al., 2012). Lastly, the fibrosis seen in our model, along with other chemical- and diet-induced murine models, is not as severe compared to human patients. Therefore, comparisons of CypI in various preclinical models despite different limitations are underway.

In summary, our results suggest that NV556 is a potent candidate for trials on NASH-derived fibrosis and HCC. Future studies include delving further into the mechanisms of action associated with the reduction of fibrosis and tumor burden as well as examining the effects of NV556 and other CypI in other NASH-driven animal models.

## Data Availability Statement

All datasets generated for this study are included in the manuscript/**Supplementary Files**.

## Ethics Statement

The animal study was reviewed and approved by the Institutional Animal Care and Use Committee of The Scripps Research Institute (La Jolla, CA) and adhered to guidelines from the National Institutes of Health (Bethesda, MD).

## Author Contributions

JK and PG designed all *in vivo* experiments. JK conducted all experiments and analyzed data. JK prepared the entire manuscript; all co-authors provided guidance on study conception and critical feedback when reviewing results and preparations of this manuscript. AG and MH synthesized and provided NV556.

## Funding

This project was funded by the National Institute of Allergy and Infectious Diseases of the National Institutes of Health under award number R01AI125365. JK is supported by the Ruth L. Kirschstein Institutional National Research Service Award (T32AI007244) at The Scripps Research Institute, sponsored by the National Institute of Allergy and Infectious Diseases. Funders had no role in study design, data collection, analysis, data interpretation, and preparation of the manuscript.

## Conflict of Interest

SS, AG, and MH are employees of NeuroVive Pharmaceutical AB. 

The remaining authors declare that the research was conducted in the absence of any commercial or financial relationships that could be construed as a potential conflict of interest.

## Abbreviations

Hepatocellular carcinoma (HCC), non-alcoholic fatty liver disease (NAFLD), non-alcoholic steatohepatitis (NASH), cyclophilins (Cyps), cyclophilin inhibitor (CypI), sanglifehrin (Sf), streptozotocin (STZ), high-fat diet (HFD).

## Supplementary Material

Figure S1 NV556 does not reduce systemic inflammation associated with NASH. Sera from STZ-injected and high-fat diet-nourished male C57BL/6J mice were detected for a series of cytokines via multiplex assay upon sacrifice. 50mg/kg NV556 or vehicle control was administered via oral gavage daily to mice for 42 days before sacrifice at 14 weeks of age. All error bars indicate ± standard error from the mean, with p-values from two-tailed Mann-Whitney test.
